# Simvastatin Protects Cardiomyocytes Against Endotoxin-induced Apoptosis and Up-regulates Survivin/NF-κB/p65 Expression

**DOI:** 10.1038/s41598-018-32376-4

**Published:** 2018-10-02

**Authors:** Lana Nežić, Ranko Škrbić, Ljiljana Amidžić, Radoslav Gajanin, Kamil Kuča, Vesna Jaćević

**Affiliations:** 1 Department of Pharmacology, Toxicology and Clinical Pharmacology, School of Medicine, University of Banja Luka, 14 Save Mrkalja St, 78000 Banja Luka, Bosnia and Herzegovina; 2 Institute of Pathology, University Clinical Center of Republic of Srpska, School of Medicine, University of Banja Luka, 12 Beba St, 78000 Banja Luka, Bosnia and Herzegovina; 3 Department of Chemistry, Faculty of Science, University of Hradec Kralove, Rokitanského 62, 500 03 Hradec Králové, Czech Republic; 4 National Poison Control Centre, Military Medical Academy, 11 Crnotravska St, 11000 Belgrade, Serbia; 5 Medical Faculty of the Military Medical Academy, University of Defense in Belgrade, 1 Pavla Jurišića-Šturma St, 11000 Belgrade, Serbia

## Abstract

This study is aimed to investigate whether simvastatin induces cardiomyocytes survival signaling in endotoxin (lipopolysaccharide, LSP)-induced myocardial injury, and if so, further to determine a role of survivin in simvastatin-anti-apoptotic effect. Wistar rats were pretreated with simvastatin (10–40 mg/kg *po*) before a single non-lethal dose of LPS. In myocardial tissue, LPS induced structural disorganization of myofibrils with significant inflammatory infiltrate (cardiac damage score, CDS = 3.87 ± 0.51, *p* < 0.05), whereas simvastatin dose-dependently abolished structural changes induced by LPS (*p* < 0.01). Simvastatin in 20 mg/kg and 40 mg/kg pretreatment, dose dependently, attenuated myocardial apoptosis determined as apoptotic index (28.8 ± 4.5% and 18.9 ± 3.5, *p* < 0.05), decreased cleaved caspase-3 expression (32.1 ± 5.8%, *p* < 0.01), along with significant Bcl-xL expression in the simvastatin groups (*p* < 0.01). Interestingly, in the simvastatin groups were determined significantly increased expression of survivin (*p* < 0.01), but in negative correlation with cleaved caspase-3 and apoptotic indices (*p* < 0.01). Simvastatin has a cardioprotective effects against LPS induced apoptosis. The effect may be mediated by up-regulation of survivin via activation of NF-κB, which leads to reduced activation of caspase-3 and consequent apoptosis of cardiomyocytes in experimental sepsis.

## Introduction

Myocardial depression is seen in approximately 50% of patients with severe sepsis, with high mortality rate as high as 70–90%^1^. A number of mechanisms have been proposed to be involved in sepsis induced myocardial dysfunction (SIMD), including bacterial toxins induced excessive production of pro-inflammatory cytokines, complement activation^2^, dysregulation of intracellular calcium transporters^3^, and mitochondrial dysfunction^4^. Subsequently, it has been suggested that functional rather than structural changes seems to be underlying mechanism of SIMD^3^. However, accumulating evidence has implicated that inflammatory infiltration and cardiomyocyte apoptosis are involved in SIMD, most likely by disruption of the myofilaments and activation of apoptosis inductors^5,6^. Gram-negative bacteria endotoxin (lipopolysaccharide, LPS) is known to induce myocardial dysfunction *in vitro*, in experimental or human sepsis, partly by excessive production of tumor necrosis factor (TNF)-α and IL-1β^5,7–9^. In sepsis induced by α-toxin of *Staphylococcus aureus* TNF-α may triggers apoptosis of rat cardiomyocyte *in vitro* or *in* vivo^7^. Moreover, during LPS- or cecal ligation and puncture (CLP)-induced sepsis, cardiomyocytes apoptosis was determined both through activation of the extrinsic and the intrinsic pathways^5^.

We and others have previously demonstrated that statins exert cholesterol-independent anti-inflammatory properties in models of acute local or systemic inflammation^9,10^, such as protection of organ tissue injuries, apoptosis of macrophages and hepatocytes, respectively^11^. Recent study showed that simvastatin has protective effects on SIMD that might be mediated by declined level of inflammatory factors TNF-a, IL-1β, IL-6, MCP-1 and.NO in myocardial tissue and serum level of cardiac troponin I^12^. Other authors showed that pretreatment with simvastatin protects against α-toxin induced sepsis that is associated with reduced proapoptotic mediators TNF-α and p53 expression, and cardiomyocyte apoptosis^7^. Nonetheless, this simvastatin-triggered cardiomyocytes survival signaling and the mechanism of its anti-apoptotic effects remain unclear.

Survivin is the smallest member of the inhibitor of apoptosis protein (IAP) family, has a dual cellular function as a regulator of cell division and an inhibitor of apoptosis. The biological functions of survivin were shown to depend on its localization, where its nuclear localization enables cell mitosis while in the cytosol it exerts anti-apoptotic activity by downregulating caspase-3 activation^13^. At present, we partially understand correlation between survivin and a vital regulator of intracellular survival pathways such as nuclear factor-kappa B (NF-κB)^14^. Accumulated evidences indicate that survivin has cardioprotective ability. Expression of survivin was induced in the myocardial infarction^13^, and heart failure^15^ in both rat and human, and also contributed to cardioprotection of insulin against myocardial ischemia/reperfusion (MI/R) injury^16^ or doxorubicin toxicity through the phophatidylinositide-3-kinase (PI3K)/Akt/mammalian target of rapamycin (mTOR) pathway^17^. However, whether survivin plays a role in simvastatin-induced cardioprotective effects against LPS is unknown.

The current study was design to determine whether pretreatment with simvastatin (1) prevents myocardial inflammatory injury and restrains apoptotic death of myocardial muscle cells, if yes (2) up-regulates survivin expression and (3) to identify the possible downstream signaling mechanism by which simvastatin regulates survivin’s expression in experimental sepsis.

## Results

### Effects of simvastatin on histopathology of cardiac tissue in experimental sepsis

**Figure 1:**
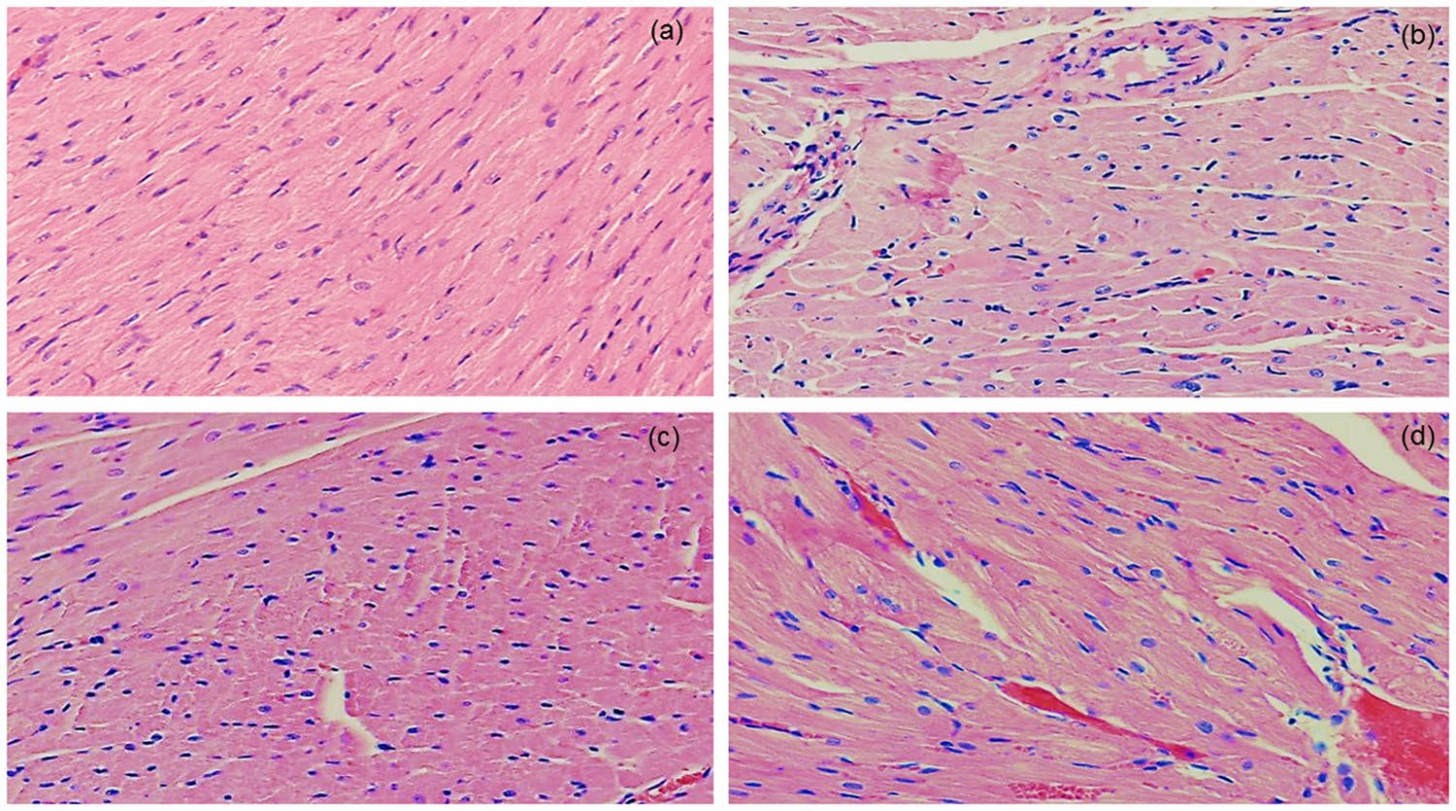
Pretreatment with simvastatin protected from LPS-induced myocardial injury. Light micrographs of the myocardial tissue, H&E, magnifications 100x and 200x). (**a**) Control group showed normal histology. (**b**) LPS group. (**c**) Simvastatin 20 group + LPS. (**d**) Simvastatin 40 group + LPS. Note decreased severe myocardial lesions and maintenance of the normal histology of the simvastatin-treated myocardium in contrast to the deterioration of myocardial tissue in the LPS-challenged group.

Microscopic examination of the cardiac tissue sections of the control animals has shown normal histological architecture with no pathological changes (Fig. 1a). Histopathologic analysis of the myocardial tissue in LPS group revealed diffuse interstitial edema, hyperemia, haemorrhages, cardiomyocyte cell degeneration and a significant cellular infiltration, predominantly polymorphonuclear leucocytes (PMNL). Small multifocal hemorrhages with perivascular infiltrate appeared widely in the cardiac tissue, accompanied with myofibrilar lysis and lost cross striations in majority myofibrils (Fig. 1b). Semiquantitative analysis of severity of cardiac lesions induced by LPS was estimated as marked with CDS = 3.87 ± 0.51 (Table 1). Pretreatment with simvastatin significantly and dose-dependently alleviated the cardiac tissue damages induced by LPS (Fig. 1c,d). Simvastatin 10 reduced the histopathological changes (CDS = 2.80 ± 0.48, not significant compared to the LPS group). As Fig. 1c shows, in the simvastatin 20 group, near-normal cardiac muscle fibers with clear cross striations are visible, but individual myofibrilar swelling, cardiomyocytes vacuolization, hyperemia and the infiltration of PMNL are significantly reduced and remained focally. The mean CDS was mild, and significantly different compared to the control group (*p* < 0.05), and to the LPS group (*p* < 0.01), respectively. Myocardial morphology in the simvastatin 40 group is mostly unchanged with mild focal hyperemia, haemorrhages, myofibril swelling, and a single PNML cells (CDS = 1.44 ± 0.43, *p* < 0.01). Semiquantitative assessment of myocardial lesions clearly reveal that simvastatin ameliorated LPS-induced cardiac tissue alterations in a dose dependent manner.

**Table 1:** Effects of simvastatin on the cardiac damage score (CDS) in LPS induced myocardial lesions in rats.

Treatment (mg/kg)	Cardiac damage score CDS 6 hearts/group × 6 slices/heart					$\bar{{\bf{x}}}$ ± S.D.
	0	1	2	3	4	
Control	36	0	0	0	0	0.00 ± 0.00
LPS	0	0	0	19	17	3.87 ± 0.54 **a**^**3**^
Simvastatin 10 + LPS	0	0	18	18	0	2.80 ± 0.48 **a**^**1**^
Simvastatin 20 + LPS	0	6	21	9	0	2.08 ± 0.65 **a**^**1**^**b**^**2**^
Simvastatin 40 + LPS	0	20	16	0	0	1.44 ± 0.43 **b**^**2**^

Statistical analysis was performed using Kruskal Wallis test. **a**^**1**^, **a**^**3**^ - *p* < 0.05, 0.001 for the results compared with the control group, **b**^**2**^ - *p* < 0.01 for the results compared with LPS group.

### Simvastatin attenuated LPS induced apoptotic cell death and inhibited cleaved caspase-3 expression in myocardial tissue

**Figure 2:**
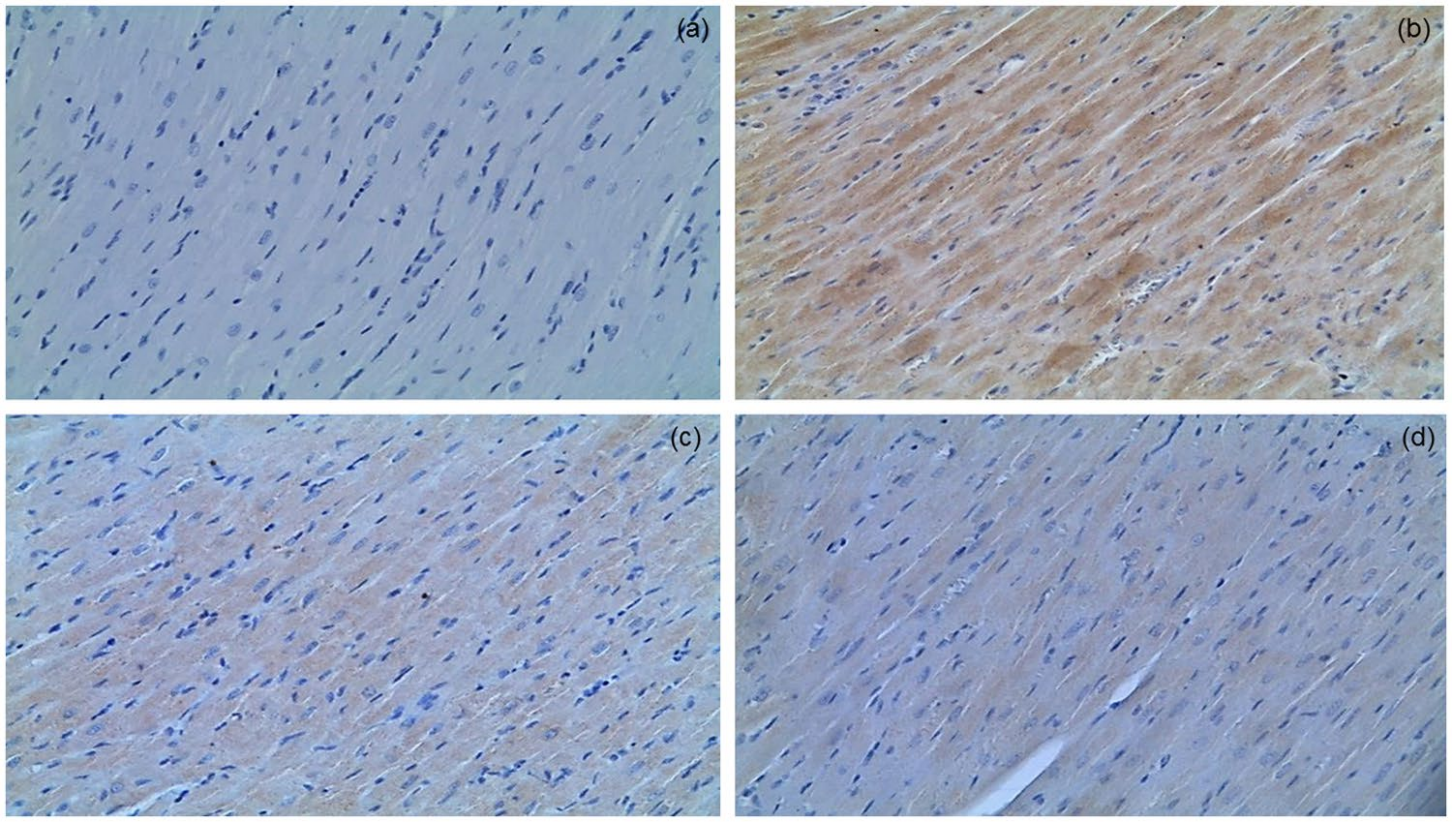
Simvastatin inhibited apoptosis in rat myocardial tissue in LPS induced inflammation by down-regulating cleaved caspase 3 expression. Representative images with quantitative analysis of apoptotic cardiomyocytes that were challenged with LPS for induction of inflammation and either pretreated with simvastatin 20 and simvastatin 40 before LPS. The expression of cleaved caspase 3 in rat myocardial tissue examined by immunohistochemical staining, magnification 200x. (**a**) Control group. (**b**) Intense cytoplasmic staining of cleaved caspase 3 in cardiomyocytes in LPS group, as characteristic of cell in apoptosis. Marked reduction of apoptotic cardiomyocytes in the simvastatin 20 (**c**) and simvastatin 40 (**d**) groups.

**Figure 3:**
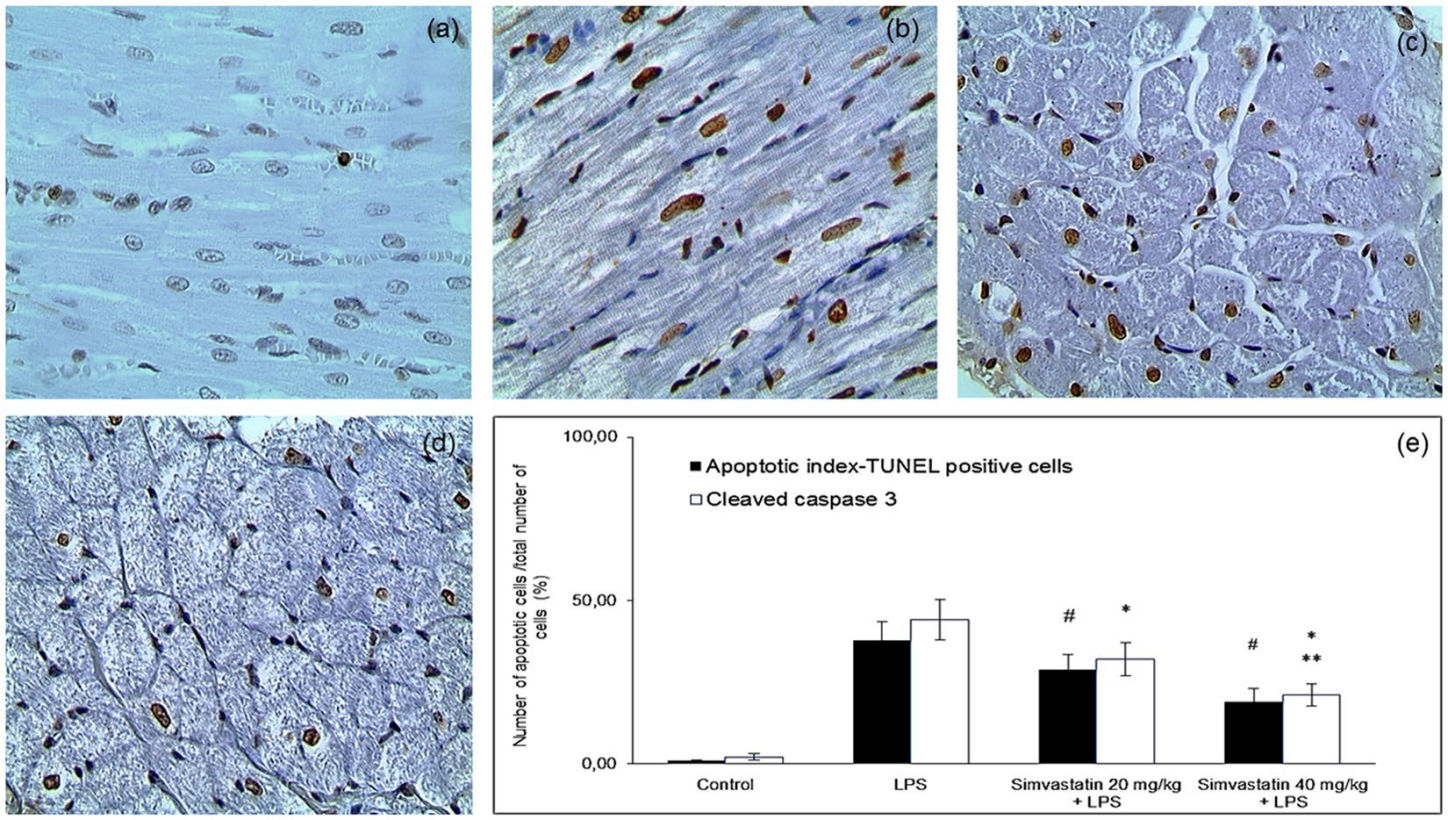
Simvastatin inhibited apoptosis in rat myocardial tissue in LPS induced inflammation detected by TUNEL staining, magnification 400x. Brown stained nuclei indicate TUNEL-positive cardiomyocytes. The apoptosis increased significantly in the LPS (**b**) and simvastatin group (**c**) compared with the control group (**a**). Note that induction of sepsis by LPS resulted in a marked appearance of TUNEL-positive cardiomyocytes (arrow) quantified and shown as AIs (black columns) (**e**), which was significantly reduced by simvastatin 20 (**c**) and simvastatin 40 (D). (**e**) Quantitative analysis of apoptotic cells counted in immunohistochemically stained myocardial sections for cleaved caspase 3 and corresponding frequencies of TUNEL positive cardiomyocytes are shown, *p < 0.01 in comparation with LPS group, **p < 0.05 in comparation with simvastatin 20, ^#^*p* < 0.05 in comparation with LPS group.

Analysis of apoptosis in myocardial tissue is given in Figs 2 and 3, including the immunohistochemically determined expression of cleaved caspase-3 and TUNEL assay. Cleaved caspase-3 is a key apoptotic molecule, and its expression is in accordance with the findings determined by TUNEL. The results showed that expression of cleaved caspase-3 in cardiomyocytes was significantly elevated in the LPS group (44.8 ± 6.2%, *p* < 0.01 compared to the control group), as well as in notable amount of cells in inflammatory infiltrate (Fig. 3b). The myocardial samples from the Simvastatin 40 group had importantly decreased incidence of cleaved caspase-3 positive muscle cells than those from the Simvastatin 20 group (20.6 ± 3.1% and 32.1 ± 5.8%, *p* < 0.05, respectively), and highly significant compared to the LPS group (*p* < 0.01) (Fig. 3c–e). Accordingly with cleaved caspase-3 findings, TUNEL assay determined a major increase of TUNEL-positive cardiomyocytes as well as the inflammatory cells due to LPS single treatment compared to a few TUNEL-stained cells in the control (Fig. 3b). However, pretreatment with simvastatin attenuated the increase of apoptosis that is observed as significantly decreased number of TUNEL-positive cardiomyocytes (Fig. 3c,d). The degree of apoptosis in myocardial tissue, expressed as the AI, increased substantially and significantly after endotoxin administration (either in LPS or simvastatin groups) compared with the control group (*p* < 0.01), but it was dose-dependently reduced in the simvastatin 20 (AI = 28.8 ± 4.5%) and simvastatin 40 group (AI = 18.9 ± 3.5%) in respect to the LPS group (AI = 37.9 ± 5.5%, *p* < 0.05, respectively) (Fig. 3e). It is important to emphasize that cleaved caspase-3 expression is detected as predominantly cytoplasmic staining, indicating apoptotic but also putative pre-apoptotic cells without chromatin condensation and with preserved cellular morphology. This could explain obvious difference in the total number of cleaved caspase-3 positive cells and apoptotic cells determined by TUNEL labeling (Fig. 3e). Nevertheless, we found there was significantly positive correlations between cleaved caspase-3 staining and TUNEL positive cells in the LPS group (R^2^ = 0.61, *p* < 0.01), the Simvastatin 20 group (R^2^ = 0.69, *p* < 0.01), and the Simvastatin 40 group (R^2^ = 0.52, *p* < 0.01), respectively.

### Changes of anti-apoptotic Bcl-xL expression in myocardial tissue after simvastatin and LPS administration

**Figure 4:**
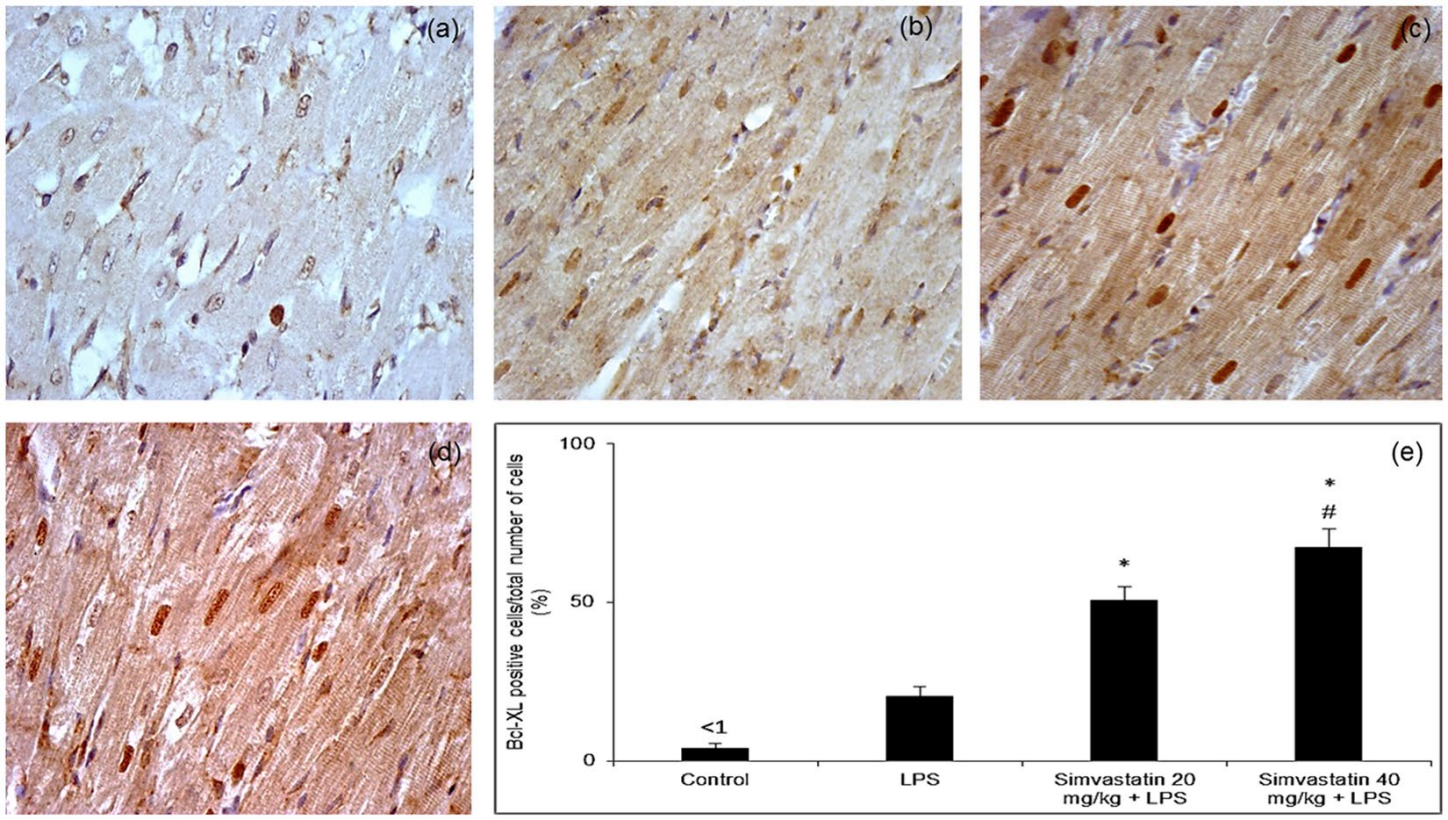
Simvastatin increased Bcl-XL expression in rat myocardial tissue in LPS induced inflammation. Representative images with quantitative analysis of distribution of Bcl-XL staining in cardiomyocytes that were challenged with LPS for induction of inflammation or either pretreated with simvastatin 20 and simvastatin 40 before LPS. The expression of Bcl-XLin rat myocardial tissue examined by immunohistochemical staining, magnification 400x. (**a**) Control group. (**b**) Note focally distributed immunopositive BCL-XL cardiomyocytes in the LPS group. In the simvastatin group 20 (**c**) and simvastatin 40 (**d**) BCL-XL expression was significantly intensive and widely distributed in cardiomyocytes. (**e**) Quantitative analysis of distribution of Bcl-XL immune-positivity cells in selected fields, *p < 0.01 in comparation with LPS group, ^#^*p* < 0.01 in comparation with simvastatin 20 group.

Immunohistochemical analysis of anti-apoptotic Bcl-xL expression in myocardial tissue showed marked difference among LPS and simvastatin groups. Results reveal weak expression of Bcl-xL in the control group (Fig. 4a), and in contrast a significant increase of the mean percentage of moderately immune-positive cardiomyocytes in the LPS group (*p* < 0.05) (Fig. 4b). Expression of Bcl-xL following LPS exposure might represent mechanism of cell protection against induced apoptosis. Pretreatment with simvastatin 20 and simvastatin 40 produced a significant increase in Bcl-xL positive cardiomyocytes with intensive cytoplasmic immune-positivity compared to the LPS group (57.8 ± 4.9% and 70.8 ± 5.2%, *p* < 0.01, respectively) (Fig. 4c–e). In the Simvastatin 40 group, we found inverse correlations between Bcl-xL immune-positive and cleaved caspase-3 (R^2^ = 0.66, *p* < 0.05), or TUNEL-positive cardiomyocytes (R^2^ = 0.58, *p* < 0.05), respectively (Figs 3e and 4e).

### Simvastatin pretreatment up-regulated survivin expression in cardiomyocytes

**Figure 5:**
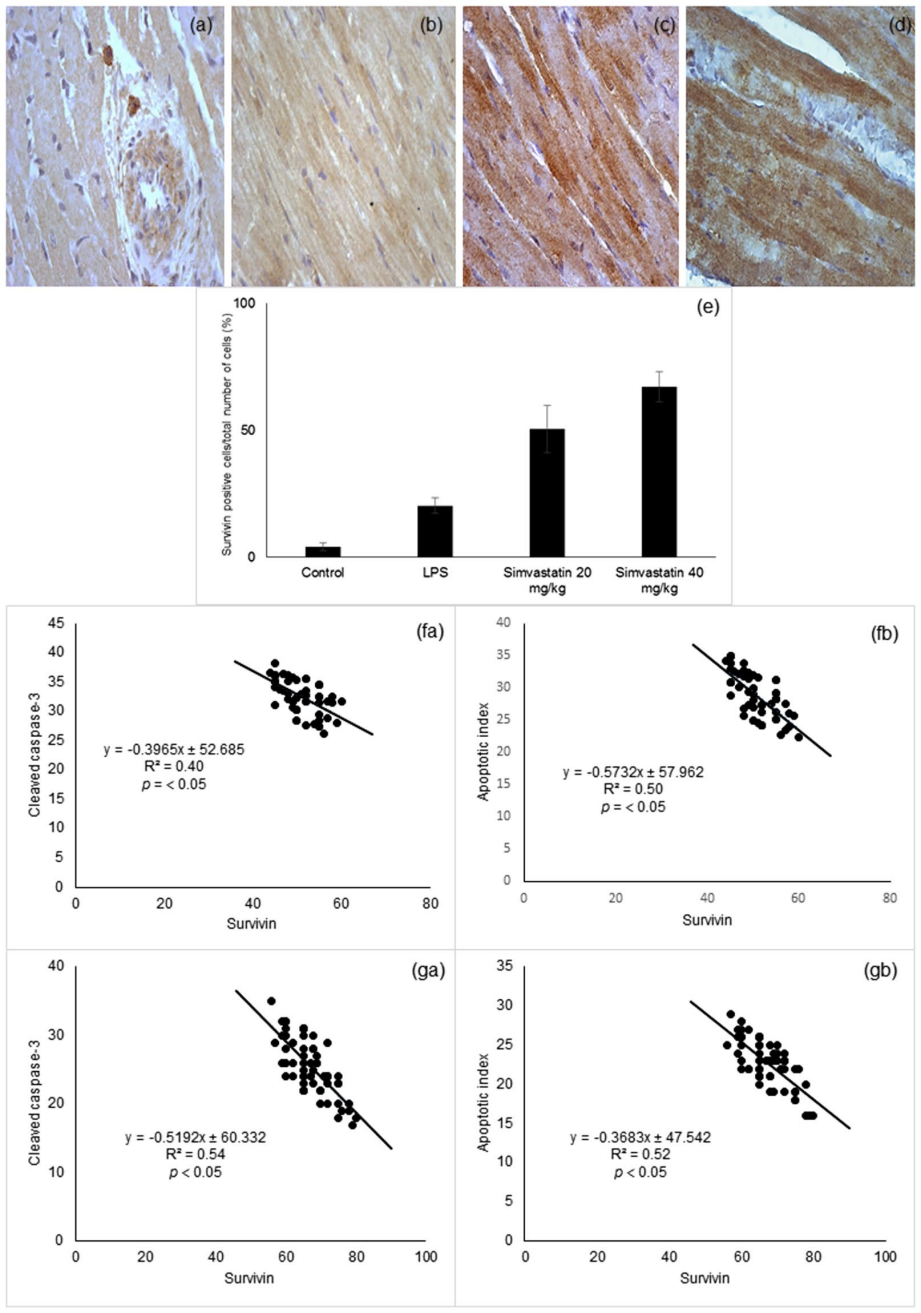
Simvastatin increased survivin expression in rat myocardial tissue in LPS induced inflammation. Representative images with semi-quantitative analysis of survivin positive cells in myocardium in groups that were challenged with LPS or either pretreated with simvastatin 20 or simvastatin 40 before LPS. Survivin expression in rat myocardial tissue examined by immunohistochemical staining, magnification 400x. (**a**) Control group. (**b**) LPS group. Note intensive survivin cytoplasmic staining of cardiomyocytes in the simvastatin 20 (**c**) and simvastatin 40 (**d**). (**e**) Semiquantitative analysis of survivin expression, *p < 0.01 in comparation with LPS group, ^#^*p* < 0.01 in comparation with simvastatin 20 group. (**f**) The correlation of survivin expression and cardiomyocytes apoptosis determined by cleaved-caspase-3 (fa) and apoptotic index (fb) in the group pretreated with simvastatin 20. (**g**) The correlation of survivin expression and cardiomyocytes apoptosis determined by cleaved-caspase-3 (ga) and apoptotic index (gb) in the group pretreated with simvastatin 40.

Basal myocardial survivin expression was barely detectable in cardiomyocytes in the control group. Endotoxin significantly elevated survivin expression levels (*p* < 0.01 compared to the control group) (Fig. 5b). Most importantly, simvastatin pretreatment significantly and dose-dependently up-regulated survivin expression demonstrated as a strong cytoplasmic staining in cardiomyocytes (Fig. 5c,d). Quantitative analysis of the extent of positive staining revealed that in the Simvastatin 20 and the Simvastatin 40 groups pretreatment led to a striking increase in survivin expression (50 ± 9.3%, and 67.2 ± 5.9%, *p* < 0.05, respectively) (Fig. 5e). As survivin, has been shown to have cardioprotective effects, we tested the correlation between survivin expression and apoptotic markers. As shown in Fig. 5f,g survivin expression are in negative correlation with cleaved caspase-3 and apoptotic index in simvastatin 20 (R^2^ = 0.40 and R^2^ = 0.50, *p* < 0.05, respectively) and 40 pretreated groups (R^2^ = 0.54 and R^2^ = 0.52, *p* < 0.05, respectively), suggesting that simvastatin protects cardiomyocytes from endotoxin by inhibiting onset of apoptosis.

### Expression of NF-κB/p65 on cardiomyocytes after simvastatin and LPS administration

**Figure 6:**
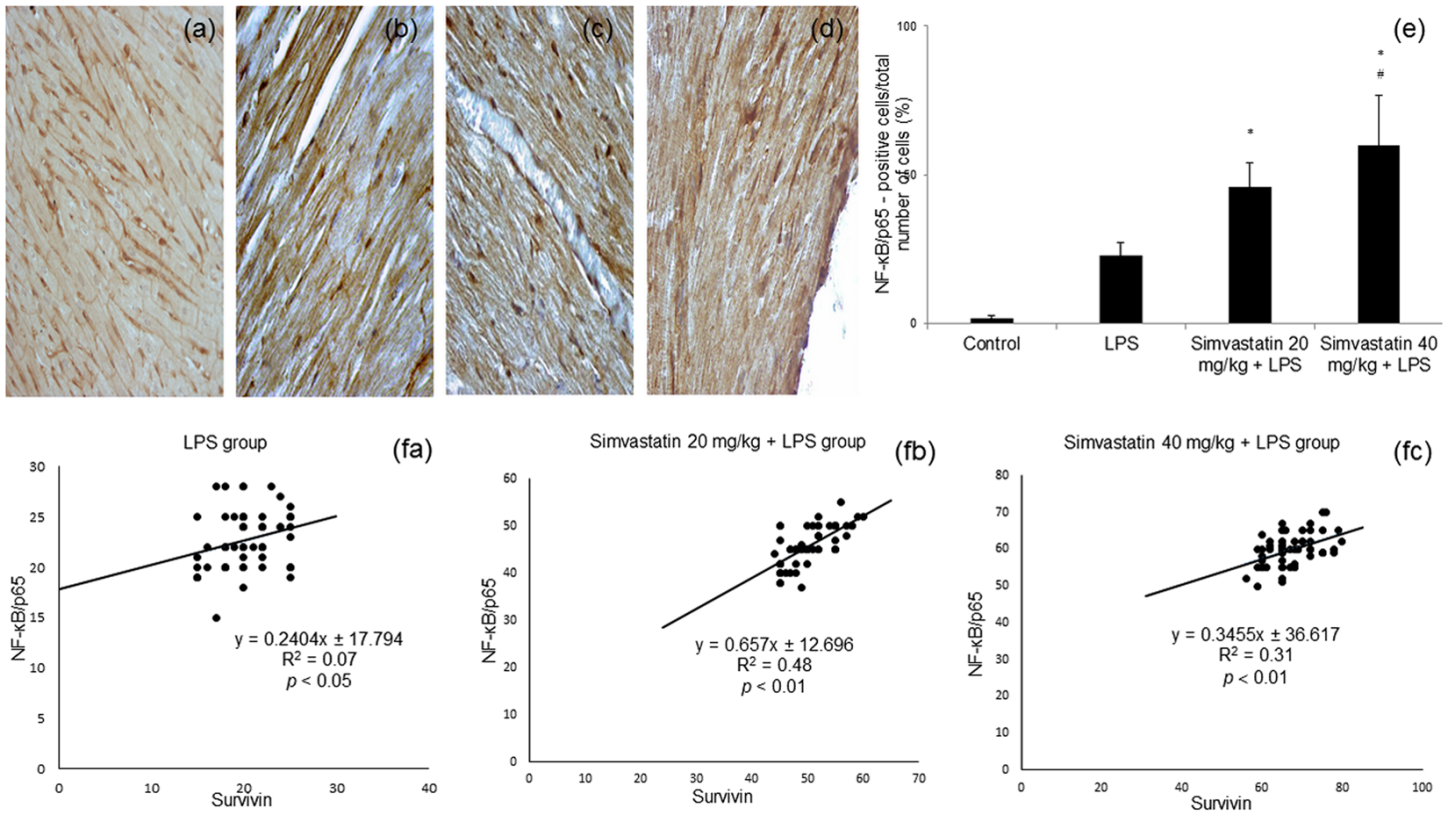
Simvastatin increased NF-κB expression in rat myocardial tissue in LPS induced inflammation. Representative images with semi-quantitative analysis of survivin positive cells in myocardium in groups that were challenged with LPS or either pretreated with simvastatin 20 or simvastatin 40 before LPS. Immunohistological staining of myocardial tissues was performed using a p65-specific antibody to evaluate NF-κB p65 expression in cardiomyocytes, magnification 200x and 400x. (**a**) Control group. Note subsets of cardiomyocytes positive for NF-κB/p65 in the cell cytoplasm and/or nucleus in the LPS group (**b**), and intensive nuclear immunostaining in the simvastatin 20 (**c**) and simvastatin 40 (**d**) groups. (**e**) Semiquantitative analysis of NF-κB/p65 expression. *p < 0.01 in comparation with LPS group, ^#^*p* < 0.05 in comparation with simvastatin 20 group. (**f**) The correlations of survivin expression and NF-κB/p65 positive cardiomyocytes within the experimental groups (fa, fb, fc).

Immunohistochemically, activation of NF-κB can be visualized by the translocation of p65 from the cytoplasm to the nucleus. As shown in Fig. 6a, in the control group immunoreactivity for NF-κB was determined as weak cytoplasmic staining in cardiomyocytes considered as basal expression. Upon LPS administration, subsets of cardiomyocytes were positive for NF-κB in the nucleus (Fig. 6b). In contrast, simvastatin pretreated groups were demonstrated a significant increase of positive NF-κB cardiomyocytes, analyzed as cells with intensive nuclear immunostaining (Fig. 6c,d) compared to LPS group (*p* < 0.05) (Fig. 6e). However, it was still unclear whether nuclear activation of NF-κB corresponds to survivin expression in cardiomyocytes in acute inflammation, which was the reason we tested correlation between their expressions within the experimental groups. Consistently, survivin expression was positively correlated with NF-κB positive cardiomyocytes in LPS group (R^2^ = 0.07, *p* < 0.05), simvastatin 20 group (R^2^ = 0.48, *p* < 0.01), and simvastatin 40 group (R^2^ = 0.31, *p* < 0.01). Those results strongly indicate on interplay between changes in survivin and NF-kB expressions following simvastatin pretreatment. However, the exact mode of survivin and NF-kB mutual interaction remains to be elucidated in the further investigations.

## Discussion

The major findings of the current study demonstrated that pretreatment with simvastatin dose-dependently prevented myocardial inflammatory injury, restrained apoptotic death of myocardial muscle cells and up-regulated expression of anti-apoptotic Bcl-xL and survivin. Myocardial survivin was also up-regulated following LPS administration, possibly acting as a self-protective mechanism along with Bcl-XL. Second, NF-kB expression was significantly increased following simvastatin pretreatment, indicating a possible downstream signaling mechanism by which simvastatin regulates survivin’s expression in experimental sepsis. These results demonstrate that survivin is a novel player in simvastatin-induced cardioprotection in experimental sepsis. Several experimental studies have demonstrated that statins prevent organ injuries against LPS or in CLP-induced sepsis^11,12,18–20^. Consistently, our results showed that recognized feature of SIMD, myocardial infiltration of predominantly polymorphonuclear leucocytes and monocytes was sensitive and significantly reduced by simvastatin. Accumulating evidences have indicated that LPS-induced cardiomyocyte apoptosis plays an important role in SIMD, as activation of caspase-3 that is a key effector of apoptosis resulted in apoptotic death, cleavage of myofilaments, and impaired contractile response of cardiomyocytes to catecholamines^21^. Our results showed that addition of simvastatin ameliorated LPS-induced cardiomyocyte apoptosis by inhibiting caspase-3 activation as well as DNA fragmentation that corresponds with the reduced myocardial injury. This effect is markedly associated with overexpressed Bcl-XL in cardiomyocytes in simvastatin groups, adding supportive data of anti-apoptotic effects of statins in limiting cell injury in a variety of septic models. Similarly, Fu *et al*. showed that simvastatin inhibited endothelial cells apoptosis in CLP model, through upregulating of anti-apoptotic Bcl-2 and downregulating pro-apoptotic Bax^22^.

Survivin is indispensable in apoptosis-regulated fetal cardio-genesis, but in the adult heart is present at low or undetectable levels^13^. However, its expression is enhanced in the peri-infarct zone and remote myocardium in human myocardial infarction^23^, is elevated in human and rat heart failure^15^, MI/R injury^16^ and in doxorubicin-induced cardiotoxicity^17^. However, weather survivin plays role in cardiomyocyte survival in experimental sepsis were remained unclear. Several authors^24^ very recently demonstrated upregulation of survivin and increased expression of thioredoxin-1, a cytosolic protein with antioxidant and anti-inflammatory properties, along with reduced apoptosis in myocardial tissue in a severe sepsis (CLP model).

Previous studies have demonstrated that in myocardial infarction and doxorubicin toxicity survivin was preferentially localized in the cytosol and that its cytoplasmic presence was able to inhibit the activation of caspase-3^13,16^. Our results have also demonstrated notable cytoplasmic survivin expression after LPS, and we assume it triggered cell-protection mechanism in the sepsis. Further, survivin in cytosol was significantly enhanced by simvastatin and inversely correlated with expression of cleaved caspase-3 and incidence of apoptotic death of cardiomyocytes confirmed by TUNEL assay. Those results strongly indicate that anti-apoptotic effects of simvastatin are at least partially survivin-dependent. However, not all IAP has to bind caspases to inhibit apoptosis. Instead, some of them activate the anti-apoptotic NF-κB/p65 or facilitate its activation by TNF-α/TNF receptor interaction^23,25^. NF-κB is found in almost all animal cells types and has a vital role in inflammation, immune response, control of apoptosis and belongs to intracellular survival pathway. The function of NF-κB is primarily regulated by IκB family members, which ensure cytoplasmic localization of the transcription factor in the resting state. Upon stimulus-induced IκB degradation, the NF-κB relocates to the nucleus and activates NF-κB-dependent transcription^26^. Also, some authors demonstrated that activation of survivin plays important role for hepatocytes against cytotoxic glycochenodeoxycholate, by activation of NF-κB that mediates hepatocytes apoptosis trough upregulation of survivin^27^. On the contrary, simvastatin suppressed proliferation of gastric cancer cells, trough inhibition of constitutive NF-kB activation, and its regulated markers of proliferation, invasion and angiogenesis including survivin^28^.

Present study demonstrates the NF-κB/p65 expression is enhanced by simvastatin, in a tightly positive correlation with survivin expression while negative with the incidence of myocardial apoptosis. These results suggest that survivin/NF-κB/p65 signaling pathway activation underlies to cardio-protective and anti-apoptotic effects of simvastatin in experimental sepsis.

In conclusion, to our best knowledge this study firstly demonstrated that the endotoxin experimental model of sepsis, a pathological cardiac stress, induced apoptosis but also triggers cell survival markers via Bcl-xL and survivin expression which can be significantly enhanced by simvastatin. Up-regulated survivin expression following NF-κB/p65 activation seems that contribute to the anti-apoptotic effects of simvastatin. These data have provided important new insights into understanding of simvastatin-induced anti-apoptotic and pro-survival actions in experimental sepsis and related organ injury stress, that may be significant basis for the clinical use of statins for SIMD, but this warrants further clinical investigation.

## Methods

### Animals

Experiments were performed on male Wistar rats, 6–8 weeks old (200 to 220 g) bred at the Department for Experimental Animals, Military Medical Academy, Belgrade, Serbia. The experimental animals were housed in groups of five in plastic cages (Macrolon® cage type 4, Bioscape, Germany) with sawdust bedding (Versele-Laga, Belgium) certificated as having contaminant levels below toxic concentrations. The environmental conditions were controlled and monitored by a central computer-assisted system with a temperature of 22 ± 20 °C, relative humidity of 55 ± 15%, 15–20 airchanges/h, and artificial lighting of approximately 220 lux (12 hrs light/dark cycle). The experimental animals had free access to food, commercial pellets for rats (Veterinarski Zavod Subotica, Serbia) and tap water from municipal mains, filtered through 1.0 μm filter (Skala Green, Serbia).

All animal care and experimental procedures were approved by (i) the Ethics Committee for Animals Experiments of the Military Medical Academy, Belgrade, Serbia (approved document 282-12/2002); (ii) all experiments were performed in accordance with Guidelines for Laboratory Animal Welfare, Ethics Committee for Animals Experiments of the Military Medical Academy, Belgrade, Serbia (decision No. 323-07-04943/2014-05/1) who was adopted in complete accordance with the current National Guidelines for Animal Welfare of the Republic of Serbia approved by the European Commission (published in the Official Gazette, Republic of Serbia, No. 41/2009).

### Drugs

Simvastatin (donated by Krka, Novo Mesto, Slovenia) was dissolved in 0.5% methylcellulose (Sigma, Taufkirchen, Germany), as 10 or 20 mg/ml stocks. Endotoxin LPS from *Escherichia coli* serotype 0127:B8 (Sigma Aldrich Munich, Germany) was injected intraperitoneally (*ip*) immediately after dilution with sterile pyrogen-free physiologic saline. All invasive procedures were operated under aseptic conditions.

### Experimental design

In this experiment, simvastatin was used in three dosing regimens (10, 20 or 40 mg/kg *per os*) that were previously shown as efficient to protect against the single median lethal dose (LD_50_) of LPS (22, 15 mg/kg *ip)* in rats^10^. Also, those doses of simvastatin are in compliance to previously employ in rat/murine studies *in vivo* (typically 10–100 mg/kg/day). On the other hand, due to significant up-regulation of HMG-CoA reductase’s activity by cause of statin treatment in rodents^11,20,21^, the same doses are higher compared to recommend for the treatment of men.

To induce experimental sepsis the animals were challenged with a non-lethal single dose of LPS *ip* (0.25 LD_50_/kg), a model that exhibits the strongest inflammatory effects in various animal models for acute systemic inflammation, including immune cell infiltration, oxidative stress and apoptosis of organ tissues^9,10,29^.

Wistar rats were randomly divided into five experimental groups each containing six individuals. The animals received the following treatments: (1) Control (0.5% methylcellulose 1 ml/kg *ip*), (2) LPS (endotoxin 5.5 mg/kg *ip*), (3) simvastatin 10 (10 mg/kg *per os*) + LPS (endotoxin 5.5 mg/kg *ip*), (4) simvastatin 20 (20 mg/kg *per os*) + LPS (endotoxin 5.5 mg/kg *ip*), and (5) simvastatin 40 (40 mg/kg *per os*) + LPS (endotoxin 5.5 mg/kg *ip*). Simvastatin was given orally *via* oral gavage for 5 days, and 1.5 h afterwards the last dose of simvastatin LPS was administered at a single dose. The animals in LPS group received the same volume (1 ml/kg) of 0.5% methylcellulose for 5 days, as a vehicle, before endotoxin injection. In the control group, an identical volume of vehicle was given, without simvastatin or LPS. After LPS administration, the animals were observed continuously for 48 hrs.

### Histological examination and semiquantitative analysis

In order to evaluate the cardioprotective effects of survivin the animals were sacrificed 48 hrs after receiving the treatment. Before sacrificing the animals, they were anesthetized with 25% urethane (4 ml/kg) (Sigma, St. Louis, USA), immobilized in a dorsal position and allowed to breathe spontaneously. After that, the cervical dislocation was used for the euthanasia of all experimental rats. At necropsy, the dissected hearth tissue were carefully spread over a metal tray coated with wax and fixed with 10% neutral buffered formalin solution. Five to seven days after fixation all tissues were divided into 4 portions in order to be prepared for making sections. After process of fixation, all tissue samples were dehydrated in graded alcohol (100%, 96% and 70%), xylol and embedded in paraffin blocks. Finally, 2-μm thick paraffin sections were stained by haematoxylin and eosin (H&E) method.

The type, degree and severity of myocardial lesions along with the degree of inflammatory cellular infiltration were assessed in tissue samples from each animal, and they were counted in six separate visual fields at x400 magnification. The severity of myocardial lesions consisting of edema, cellular infiltration, hemorrhages, myofibrilar vacuolar degeneration, myofibrilar lysis, and the distribution of lesions (e.g., focal, multifocal, locally extensive, or diffuse) were assessed and graded by two independent pathologists. From each slices, whole visual fields were analyzed by using light microscope according to the 5-point semiquantitative scale (0 = no change, 1 = minimal, 2 = mild, 3 = moderate, and 4 = marked) according to the degree and extent of the changes described above^22,30^. A severity grade, expressed as cardiac damage score (CDS) of myocardial lesions, was determined for the right ventricle, left ventricle, and septum, and the mean CDS was determined (Table 1).

### ***In situ*** determination of apoptosis in rat myocardial tissue-TUNEL method

Apoptosis at a cellular level was assessed by means of Terminal deoxynucleotidyl transferase mediated dUTP Nick End Labeling (TUNEL) method. An *In Situ* Cell Death Detection Kit POD (Roche Molecular Biochemicals, Switzerland, Cat. No. 11 684 817 910) was used to carry out TUNEL staining on paraffin-embedded sections of 4–6 μm thickness according to the manufacturer’s instructions.

Tissue sections were incubated with anti-fluorescein antibody conjugated with horse-radish peroxidase (POD), and then color development was performed using diaminobenzidine (DAB) substrate. Negative (incubation with Label Solution, instead of TUNEL reaction) and positive controls (incubation with DNase I recombinant, grade I) were performed per the manufacturer’s instructions. Immuno-labeled (TUNEL positive) cells were estimated by single-blinded assessment. The slides were examined under a light microscope (Olympus, Tokyo, Japan) at x400 magnifications. Twenty non-successive fields per sample were counted for the number of TUNEL-positive cardiomyocytes. The percentage (%) of apoptotic cardiomyocytes expressed as an apoptotic index (AI) was calculated according to the formula:

$$\begin{array}{c}{\rm{AI}}( \% \,{\rm{of}}\,{\rm{apoptotic}}\,{\rm{cells}})={\rm{the}}\,{\rm{number}}\,{\rm{of}}\,{\rm{TUNEL}}-{\rm{positive}}\\ \,\,\mathrm{cardiomyocytes}/\mathrm{the}\,{\rm{total}}\,{\rm{number}}\,{\rm{of}}\,{\rm{cardiomyocytes}}\,\times \,100\end{array}$$

### Immunohistochemical determination of apoptosis–regulating molecules

Paraffin-embedded sections of myocardial tissue were stained with a polyclonal rabbit antibodies for pro-apoptotic cleaved (activated) caspase-3 (Asp 175) (9661, Cell Signaling Technology, Frankfurt, Germany), and anti-apoptotic Bcl-xL, member of the Bcl-2 family of proteins (PA1–37161, Scientific Pierce Product, Rockford, USA), survivin monoclonal mouse antibodies for survivin clone 8E2 (MS-1201-P1 NeoMarkers Fremont, CA, USA), and NF κB/p65 (RB-1638-R7 NeoMarkers Fremont, CA, USA), according to the manufacturer’s instructions. Briefly, for immunohistochemical analysis 3–4 μm tissue sections were deparafinated and rehydrated. Slides were then boiled for 20 minutes in a microwave oven with citric acid buffer solution (0.01 mol/L citrate buffer, pH 6.0.). To reduce nonspecific background staining, slides were incubated in 3% hydrogen peroxide for 10 minutes. Primary antibodies for cleaved caspase-3 (1:300), Bcl-xL (RTU), survivin (1:50) and NF-κB/p65 (RTU) were applied according to manufacturer’s recommended protocol. The slides were washed thoroughly with phosphate buffered saline (pH 7,4) between the steps. 3,3′-Diaminobenzidine (DAB) (TL-015-HDJ, Thermo Scientific Lab Vision UltraVision ONE Detection System) was used as chromogen, to develop the antigen-antibody complex, and all slides were afterward counterstained with H&E, dehydrated, and mounted. Appropriate positive and negative controls were processed in parallel. The slides were examined under a light microscope (Olympus, Tokyo, Japan) at x200 and x400 magnification. Twenty non-successive fields per sample were counted for the number of color-positive cells by two pathologist as single-blinded assessment, using ImageJ software 1.50. Survivin expression in cardiomyocytes were evaluated qualitatively, where cells positive for cytoplasmic staining were considered immunopositive and taken into account^16^. Although cytoplasmic expression of NF-κB/p65 is detected in most normal cells, only distinct brown nuclear immunostaining (brown granules in the nucleus) is considered as activated NF-κB/p65, and quantified as previously described^31,32^. The number of survivin or NF-κB/p65 positive cardiomyocytes was expressed as a percentage (%) according to this formula:

$$\begin{array}{c}{\rm{Percentage}}\,{\rm{of}}\,{\rm{survivin}}\,{\rm{or}}\,{\rm{NF}}-{\rm{\kappa }}B/{\rm{p65}}\,{\rm{positive}}\,{\rm{stained}}\,{\rm{cells}}( \% )\\ \,\,\,=\,{\rm{the}}\,{\rm{number}}\,{\rm{of}}\,{\rm{positively}}\,{\rm{stained}}\,{\rm{cardiomyocytes}}/\\ \,\,\,\,{\rm{total}}\,{\rm{number}}\,{\rm{of}}\,{\rm{cardiomyocytes}}\times 100\end{array}$$

### Statistical analysis

All the data are presented as the mean ($\bar{{\rm{x}}}$) ± standard deviation (S.D.). The differences in TDS between groups were compared using the Kruskal–Wallis rank test. The differences in biomarkers expression among groups were compared by analysis of variance (ANOVA) followed by the Tamhane’s T2 *post-hoc* test, and correlation analysis was performed with Pearson’s correlation coefficient. A value *p* < 0.05 was considered statistically significant. The experimental data were analyzed using SPSS 19.0 statistical software.
